# Effect of laminar air flow and building construction on aspergillosis in acute leukemia patients: a retrospective cohort study

**DOI:** 10.1186/s12879-018-3665-9

**Published:** 2019-01-09

**Authors:** Makoto Iwasaki, Junya Kanda, Masakatsu Hishizawa, Toshiyuki Kitano, Tadakazu Kondo, Kouhei Yamashita, Akifumi Takaori-Kondo

**Affiliations:** Department of Hematology and Oncology, Graduate School of Medicine, Kyoto University, 54 Kawaharacho, Shogoin, Sakyo-ku, Kyoto, 606-8507 Japan

**Keywords:** Aspergillosis, Acute leukemia, Laminar air flow, Prevention, Fungal infection

## Abstract

**Background:**

The preventive effect of laminar air flow (LAF) on aspergillosis has been observed in patients with hematological malignancies. However, the short follow-up period limits the interpretation of study results.

**Methods:**

To assess the preventive effect of long-term LAF use on aspergillosis in its long-term use, we retrospectively analyzed 124 acute leukemia patients at our hospital between January 2005 and March 2016. We compared the incidence of aspergillosis before (May 2008) and during the construction of a new building (June 2008–January 2010) and in the early (February 2010–March 2014) and late (April 2014–March 2016) periods after moving to a new hematology ward with an LAF system. The 2008 European Organization for Research and Treatment of Cancer and Mycosis Study Group criteria were used for the diagnosis of aspergillosis.

**Results:**

Fourteen patients were diagnosed with possible, probable, or definite aspergillosis. Cumulative incidence rates of aspergillosis at day 180 were 12.4, 24.9, 9.3, and 25.1% before construction, during construction, in the early period after moving to a new ward, and in the late period after moving to a new ward, respectively (*p* = 0.106). Multivariate analysis showed that the LAF system tended to reduce the risk of aspergillosis in the early period (before construction vs. early period; hazards ratio (HR) = 1.97, *p* = 0.463 and during construction vs. early period;HR = 3.42, *p* = 0.184), but the risk increased in the late period (late vs. early period, HR = 5.65, *p* = 0.035).

**Conclusions:**

Building construction might increase the risk of aspergillosis. Short-term LAF use might reduce aspergillosis risk, but its long-term use is inadequate, although we could not exclude the possibility of increased risks in the recent period due to continued improvements in the different areas of our hospital. Strict maintenance, more effective LAF system, and optimization of aspergillosis prophylaxis may be necessary.

## Background

Although the fungal disease-related mortality rate in acute leukemia patients has decreased with the development of effective antifungal drugs and early intervention, mortality remains high [1, 2]. Exposure to fungi during the pre-hospitalization and hospitalization period is an important risk factor for fungal infection [2, 3]. Therefore, avoidance of fungal exposure is essential, and one of the effective procedures used to prevent fungal infection, especially mold infections such as invasive aspergillosis, is laminar air flow (LAF) isolation. Building construction or demolition of an adjacent ward area was reported to be associated with an increase in fungal spores in the ward and to be one of the important risk factors for outbreaks of invasive aspergillosis [3, 4]. LAF systems have been reported to be effective in the prevention of invasive aspergillosis; they are also reported to be cost-effective [3–5]. Although these reports showed the effectiveness of LAF in the early period after the construction of wards, the long-term preventive effect of LAF isolation has not been assessed sufficiently. Therefore, in the present study, we aimed to conduct a retrospective analysis of the incidence of invasive aspergillosis in newly diagnosed acute leukemia patients treated in our hospital and assess the preventive effect of long-term LAF isolation against invasive aspergillosis.

## Methods

### Data collection

We included patients who were newly diagnosed with acute leukemia and hospitalized in the Hematology and Oncology department of the Kyoto University Hospital and who received a series of induction and consolidation chemotherapies between January 2005 and March 2016. We excluded patients for whom data on survival status, treatment regimen, antifungal prophylaxis, and infection status were lacking; those who had already been diagnosed with invasive fungal disease; those who had received antifungal treatment on initiation of chemotherapy; and those who did not receive intensive treatment, i.e. those who received supportive care or reduced-intensity chemotherapy. A total of 124 acute leukemia patients were included in this study.

### Isolation strategies

In the period before and during construction, all the patients were treated in a ward without an LAF system. In the period after moving, all patients were treated in a ward with an LAF system. In line with the International Organization for Standardization (ISO) 14,644–1; 2015 classification, we periodically examined the cleanliness of the ward to confirm that all the ward areas conformed to ISO 7–8 (class 10,000–100,000 in clean room classification according to US guidelines) and the inpatient rooms conformed to ISO 6 (class 1000, for induction chemotherapy) or ISO 7 (class 10,000, for consolidation chemotherapy). We encouraged patients to rehabilitate outside their rooms even if they had neutropenia, and if the absolute neutrophil count was > 200 cells/mm^3^ for 2 days, the patient was permitted to go outside the ward in the period after moving to an LAF-containing ward.

### Prophylaxis of fungal infection

Fluconazole was used as first-line antifungal prophylaxis for induction and consolidation chemotherapy of acute leukemia. If patients could not receive drugs orally, micafungin was used as an alternative prophylactic agent. When the LAF system malfunctioned in January 2013, micafungin was used as the first-line prophylactic agent to prevent aspergillosis until July 2013. In two patients, itraconazole was used for prophylaxis as ordered by the attending physician. In some cases, empirical therapy with mould-active antifungal agent, such as echinocandins, voriconazole, or amphotericin B was required for treating antibiotic-resistant febrile neutropenia. Empirical use of antifungal drugs without diagnosis of fungal infection was considered for preventive use.

### Endpoints

The primary endpoint of this study was the incidence of aspergillosis. We compared the incidence of aspergillosis before (May 2008, phase A) and during the construction of a new building (June 2008–January 2010, phase B) and in the early (February 2010–March 2014, phase C) and late period (April 2014–March 2016, phase D) after moving to the new hematology ward with an LAF system. The other endpoint that we assessed was the overall incidence of fungal infection. The 2008 European Organization for Research and Treatment of Cancer and Mycosis Study Group criteria (EORTC/MSG 2008 criteria) were used for the diagnosis of aspergillosis. Without microbiological or histopathological findings, patients were diagnosed with aspergillosis when lung involvement was detected on radiographical examinations, such as computed tomography, and patients with findings suggestive of fungal infections other than lung involvement and those who had no microbiological and histopathological findings were diagnosed with fungal infection, but not with aspergillosis. The day of diagnosis was defined as the day when the criteria for possible, proven, or probable aspergillosis or fungal infection were fulfilled.

### Statistical analysis

Descriptive statistics were used to summarize patient characteristics. Comparisons among groups were performed with the Fisher’s exact test for categorical variables and the Kruskal-Wallis test for continuous variables. The incidence of aspergillosis was estimated on the basis of cumulative incidence and was compared using the Gray’s test. Competing events were death without aspergillosis and the incidence of fungal infections other than aspergillosis. Cox proportional hazards ratio was used to evaluate the effect of the confounding covariates, including time-dependent covariates. We also performed Fine and Gray proportional hazards regression analysis to consider competing events but without including time-dependent covariates.

The following variables were considered in the univariate and multivariate analyses: period of neutropenia, lineage of leukemia, age, LAF isolation usage, and exposure to construction (i.e. comparison between before construction, during construction, and the early and late periods after moving to the new ward). Period of neutropenia (≤60 vs. > 60 days) was treated as a time-dependent covariate because the risk of fungal infection increased with the level of neutropenia and repeated chemotherapy. A *p* value of < 0.05 was used to determine statistical significance.

All statistical analyses were performed with R (the R Foundation for Statistical Computing, version 3.1.1, Vienna, Austria) and EZR (Saitama Medical Center, Jichi Medical University, Saitama, Japan) [6].

## Results

### Patient characteristics

Patient characteristics are summarized in Table 1. Median follow-up period was 139 days, and the median age at diagnosis was 52 years (range, 15–75 years). A total of 72 men and 52 women were included in this study.

**Table 1:** Patient characteristics

Characteristics	whole period	phase A	phase B	phase C	phase D	*p* value
sex						
female	52	16	12	17	7	0.059
male	72	20	6	26	20	
age (years)	15–75 (52.0)	25–73 (56.5)	19–71 (46.5)	18–75 (51)	15–70 (52)	0.763
> median(53–75)	59	19	8	19	13	0.890
< median(15–52)	65	17	10	24	14	
neutropenia (days)	12–179 (61)	15–150 (65.5)	12–168 (57.0)	18–122 (58.0)	12–179 (53.0)	0.520
> median(62–179)	60	22	8	19	11	0.342
< median(12–61)	64	14	10	24	16	
lineage						
AML	102	31	15	33	23	0.729
ALL	22	5	3	10	4	
prophylaxis						
FLCZ only	69	22	14	22	11	0.082
mold-active agent	55	14	4	21	16	
ITCZ	6	3	0	1	2	
VRCZ	5	1	1	1	2	
MCFG	50	12	3	21	14	
CPFG	1	0	0	0	1	
AMB	1	1	0	0	0	
L-AMB	5	0	1	3	1	
treatment response after induction chemotherapy						
CR	76	21	11	24	20	0.677
CRi	3	1	1	1	0	
non-CR	45	14	6	18	7	
median follow-up of event free survivors (days)	139.0 (27–330)	132.5 (29–236)	149.0 (27–330)	139.0 (42–261)	135.0 (41–272)	0.963

Note. AML denotes acute myelogenous leukemia; *ALL* acute lymphoblastic leukemia, *FLCZ* fluconazole, *ITCZ* itraconazole, *VRCZ* voriconazole, *MCFG* micafungin, *CPFG* caspofungin, *AMB* amphotericin b, *L-AMB* liposomal amphotericin b, *CR* complete remission, *CRi* complete remission with incomplete hematologic recovery

In the late period after moving, patients often received mold-active antifungal agents, such as itraconazole, voriconazole, micafungin, caspofungin, amphotericin B, and liposomal amphotericin B; in the construction period, patients who often received only fluconazole for prevention; however, the difference between the two groups was not statistically significant. Although we used micafungin as the first-line prophylaxis due to trouble in LAF system functioning during a certain period that fell within the early period after moving, usage of mold-active agenst did not increase in that period.

Details about acute leukemia subtypes and treatment strategies are summarised in Table 2. More number of patients were treated using intensive high-dose cytarabine based regimens was higher in the period before construction than in the other periods. All eight patients with Philadelphia chromosome positive acute lymphoblastic leukemia or chronic myeloid leukemia were treated by chemotherapy with tyrosine kinase inhibitors such as imatinib or dasatinib. Of the six patients with T-cell acute lymphoblastic leukemia after moving (Phase C or D), four patients were treated with nelarabine containing regimens; the other two patients with early T-cell precursor acute lymphoblastic leukemia were treated with standard-dose cytarabine and anthracycline.

**Table 2:** Description about leukemia subtypes and treatment strategies

Characteristics	whole period	phase A	phase B	phase C	phase D	*p* value
AML subtype						
de novo AML	49	13	6	17	13	0.646
AML-MRC	33	13	5	8	7	
t-MN	11	3	1	6	1	
APL	5	1	2	1	1	
CML-BC	1	1	0	0	0	
ALL subtype						
B-ALL	10	4	0	6	0	0.092
T-ALL	8	0	2	3	3	
CML-BC	3	0	1	1	1	
induction treatment for AML						
SDAC with anthracycline^a^	86	21	11	31	20	0.002
HDAC based treatments^a^	11	9	1	0	1	
anthracycline with ATRA	5	1	2	1	1	
induction treatment for ALL						
HyperCVAD-MA	17	5	4	8	0	0.002
other treatments	5	0	0	3	5	

AML denotes acute myelogenous leukemia; AML-MRC AML with myeloid dysplasia related changes, *t-MN* treatment related myeloid neoplasia; *APL* acute promyelocytic leukemia, *CML-BC* chronic myeloid leukemia blast crisis, *ALL* acute lymphoblastic leukemia, *Ph-ALL* Philadelphia chromosome positive ALL, *B-ALL* B cell ALL, *T-ALL* T cell ALL, *SDAC* standard-dose cytarabine, *HDAC* high-dose cytarabine, *ATRA* all-trans retinoic acid, HyperCVAD-MA hyperfractionated cyclophosphamide, vincristine, doxorubicin, and dexamethasone alternating with high-dose methotrexate, and cytarabine ^a^SDAC means 100-200mg/ m2 of cytarabine and HDAC means more than 1-2g/ m2 of cytarabine

### The incidence of fungal infection

**Fig. 1:**
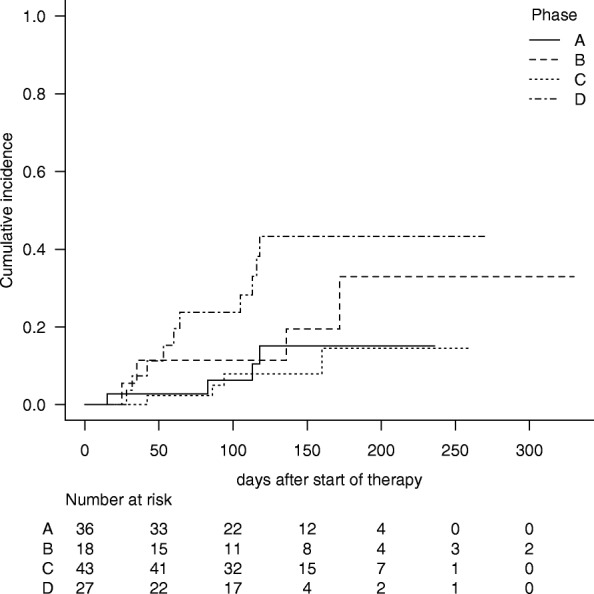
Cumulative incidence of fungal infection. Cumulative incidence of fungal infection in phase A (before construction; the solid line), B (during construction; the long dotted line), C (in the early period after moving to a new ward; the short dotted line) and D (in the late period after moving to a new ward; the dashed dotted line)

Twenty patients were diagnosed with possible, probable, or definite fungal infection (Table 3). However, two patients could not be diagnosed with fungal infection according to the EORTC 2008 criteria; however, one of the two patients had exudative plaques in the fundus, suggesting candidiasis, and the other patients did not show positive radiographical, microbiological or histopathological findings of fungal infection but had prolonged febrile neutropenia with continuously increasing β-d-glucan values. As a result, 22 patients (17.7% of the participants) were included in the fungal infection group. The cumulative incidence rates of fungal infection at day 180 were 15.1, 32.9, 14.4, and 43.3%, in phase A, B, C, and D, respectively (Fig. 1, Gray test, *p* = 0.014). Multivariate analysis showed that the risk increased significantly in the late period after moving (phase D vs. phase C; hazard ratio (HR) = 4.63, *p* = 0.010) (Table 4). Fine-Gray proportional hazards regression for competing events showed significantly higher risk in the late period than in the early period after moving (phase D vs. phase C; HR = 4.64, *p* = 0.0083) (Table 4).

**Table 3:** Incidence of fungal infection

Characteristics	whole period	phase A	phase B	phase C	phase D	*p* value
fungal infection	22 (17.7%)	4 (11.1%)	4 (22.2%)	4 (9.3%)	10 (37.0%)	0.022
none	102	32	14	39	17	
possible	14	3	3	4	4	
probable	4	0	1	0	3	
proven	2	1	0	0	1	
suspect of^a^	2	0	0	0	2	
aspergillosis	14 (11.3%)	3 (8.3%)	3 (16.7%)	2 (4.7%)	6 (22.2%)	0.103
none	110	33	15	41	21	
possible	10	3	2	2	3	
probable	4	0	1	0	3	

^a^Two patients could not be diagnosed with fungal infection according to the EORTC 2008 criteria but had clinical findings that were highly suggestive of fungal infection

**Table 4:** Multivariate analysis on the incidence of fungal infection

variables	Adjusted HR with model 1^a^	*p* value	Adjusted HR with model 2^b^	*p* value
Neutropenia	0.74	0.647		
treatment period^c^				
before construction (phase A)	1.32	0.698	1.21	0.790
during construction (phase B)	2.75	0.163	2.82	0.140
the late period after moving (phase D)	4.63	0.010	4.64	0.008
antifungal prophylaxis^d^	1.40	0.702	1.41	0.430
ALL vs AML	0.59	0.423	0.62	0.470
age	1.19	0.717	1.23	0.660

AML denotes acute myelogenous leukemia; *ALL* acute lymphoblastic leukemia, *HR* hazard ratio

^a^Model 1 is a Cox proportional hazards ratio with time-dependent covariates

^b^Model 2 is a Fine and Gray proportional hazard regression model

^c^HR is calculated by comparison of the incidence in each period with in the early period after moving

^d^HR is the ratio of the incidence in patients who received fluconazole with mold-active agent prophylaxis with the incidence in patients who received fluconazole-only prophylaxis

### Incidence of aspergillosis

**Fig. 2:**
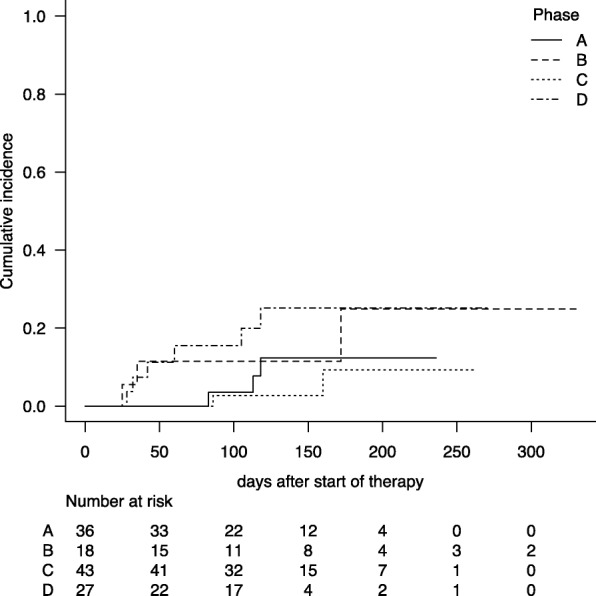
Cumulative incidence of aspergillosis. Cumulative incidence of aspergillosis in phase A (before construction; the solid line), B (during construction; the long dotted line), C (in the early period after moving to a new ward; the short dotted line) and D (in the late period after moving to a new ward; the dashed dotted line)

Fourteen patients (11.3% of the participants) were diagnosed with possible, probable, or definite aspergillosis according to the EORTC/MSG 2008 criteria (Table 3). As shown in Fig. 1, the cumulative incidence rates of aspergillosis at day 180 were 12.4, 24.9, 9.3, and 25.1% in phase A, B, C, and D, respectively (Fig. 2, Gray test, *p* = 0.106). Multivariate analysis with time-dependent covariates showed that use of LAF systems tended to reduce the risk of aspergillosis in the early period (phase A vs. phase C; HR 1.97, *p* = 0.463 and and phase B vs. phase C;HR = 3.42, *p* = 0.184), but that the risk was increased in the late period after moving (phase D vs. phase C; HR = 5.65, *p* = 0.035) (Table 5). Fine-Gray proportional hazards regression for competing events showed the same increasing tendency in the late period after moving (phase D vs. phase C; HR = 5.28, *p* = 0.030) (Table 5).

**Table 5:** Multivariate analysis of the incidence of aspergillosis

variables	Adjusted HR with model 1^a^	*p* value	Adjusted HR with model 2^b^	*p* value
Neutropenia	0.45	0.362		
treatment period^c^				
before construction (phase A)	1.97	0.463	1.76	0.540
during construction (phase B)	3.42	0.184	3.60	0.170
the late period after moving (phase D)	5.65	0.035	5.28	0.030
antifungal prophylaxis^d^	0.88	0.818	0.82	0.800
ALL vs AML	0.46	0.348	0.56	0.480
age	0.81	0.709	0.86	0.690

AML denotes acute myelogenous leukemia; ALL, acute lymphoblastic leukemia; HR, hazard ratio

^a^Model 1 is a Cox proportional hazards ratio model with time-dependent covariates

^b^Model 2 is a Fine and Gray proportional hazard regression model

^c^HR is calculated by comparison of the incidence in each period with in the early period after moving

^d^HR is the ratio of the incidence in patients who received fluconazole with mold-active agent prophylaxis with the incidence in patients who received fluconazole-only prophylaxis

## Discussion

Fungal infection is a serious complication in patients with hematological malignancies who are severely immunocompromised in its nature and by intensive treatment, such as chemotherapy or hematopoietic stem cell transplantation (HSCT) [1, 2]. Some fungi are spread through air transmission, and the most common airborne fungus in immunocompromised hosts is aspergillus. Environmental control is very essential in the preventing infections caused by airborne fungi, like aspergillosis and a widely used prevention method is use of the LAF isolation system.

The LAF system is generally used for protective isolation of immunocompromised patients, especially those with hematological malignancies receiving intensive chemotherapy or undergoing HSCT and having prolonged neutropenia. Previously, the LAF isolation system has been shown to be effective in preventing mold fungal infections and a large case series of HSCT-treated patients suggested that this protective effect against mold fungal infections could improve treatment-related mortality (TRM) [7]. By contrast, a recent report showed that HSCT could still be performed in outpatient settings that had an invasive aspergillosis incidence as low as that in inpatient settings [8]. This discrepancy is caused by multiple factors, such as patient background, treatment strategy, and local environmental factors (fungi prefer warm and humid climate); thus, we should reconsider when and how LAF isolation systems can be used efficiently.

In the present study, we investigated the effectiveness of the LAF isolation system after long-term use during the construction period in an adjacent area of the hospital, and we showed that the protective effect of the LAF isolation system against fungal infection decreased after long-term use especially with respect to airborne fungi infections like aspergillosis. This result suggested that more efforts should be made for establishing protective isolation systems, including LAF system maintenance and more intensive preventive strategies should also be developed against fungal infections with long-term use of LAF.

In terms of protective isolation, construction in the adjacent ward might have increased aspergillosis incidence in the late period after LAF ward construction. Many reports showed that construction, renovation, demolition, and excavation of buildings around the hospital are reported to be an important risk factors for fungal infection, and the most important pathogen in this setting is aspergillus [2–4, 9, 10]. Previous reports showed that the LAF systems could overcome aspergillosis outbreaks by inhibiting environmental exposure of fungal spores, but these reports did not investigate the effectiveness of LAF after long-term use [3, 4, 11].

The Centers for Disease Control and Prevention provided the Guidelines for Environmental Infection Control in HealthCare Facilities, which claimed the importance of maintenance of air handling systems, infection control risk assessment, and isolation from dusty external areas [12]. However, a detailed standard is needed for assessment and development of maintenance strategies with respect to air quality.

Some reports have shown that the risk of fungal infection can be partially predicted by counting fungal spore colony-forming units [4, 10]. Although the ward area’s conformance to the ISO criteria was checked, aspergillus spores are 3–10 μm in diameter. Hence, some pathogenic spores might have existed especially outside the rooms in the ward area during the construction of the adjacent ward.

A recent report showed that environmental control measures other than LAF isolation systems, during construction were effective in preventing invasive aspergillosis [13]. These environmental control measures include dust control procedures and barriers during construction, and education of healthcare workers regarding the dangers of nosocomial invasive aspergillosis. We should reconsider these simple and fundamental approaches for maintaining hygiene even under LAF isolation settings and for development of maintenance procedures for wards with the LAF isolation systems.

We should also reconsider the times at which patients are permitted to go outside the rooms or the ward. Prolonged neutropenia is an important risk factor of fungal infections, but environmental control strategies according to neutrophil counts usually vary with each institution. The SEIFEM 2008 registry study showed that neutrophil count was usually below 500 cells/mm^3^ at the onset of aspergillosis [1]. Hence, the threshold of neutrophil count should be defined; patients with neutrophil counts above this threshold value are safe go outside the ward with respect to airborne infections, including aspergillosis. A previous report showed that introduction of high-efficiency filtration masks that filter particles sized 0.1 μm prevented aspergillosis efficiently in neutropenic patients when they went outside the ward with LAF isolation [14]. Introduction of high-efficiency filtration masks to neutropenic patients when they go outside the ward with LAF isolation or even when they go outside their rooms to rehabilitate might be a promising strategy.

From the view point of the preventive strategy other than air quality maintenance, some mold-active antifungal agents could prevent fungal infection, but it is still controversial whether these agents should be introduced as prophylactic treatments [15–17]. A previous study reported that posaconazole was superior to fluconazole and itraconazole in the incidence of fungal infection and its mortality at day 100 after hematopoietic stem cell transplantation. Another report showed that voriconazole and caspofungin are promising agents for preventing aspergillosis during the construction of the areas adjacent to the ward [15]. However, the superiority of voriconazole to fluconazole has not been proven in the previous randomized controlled trials [16]. Because this study was conducted in 35 centers, heterogeneity in environmental exposure may be observed. Hence, the recommended antifungal agents for patients who are highly exposed to environmental factors, such as construction, renovation, and long-term use of the LAF systems after its introduction, should be further studied.

Our study had some limitations, including the small study cohort and non-randomized and retrospective study design. Currently, the LAF isolation system is used for the prevention of mold infection to such an extent that randomized-controlled study design is not pragmatic due to ethical considerations. In this study, we used EORTC/MSG 2008 criteria for diagnosis of fungal infection in this study, but we also included two patients who were highly suspected of fungal infection by the attending physicians without being diagnosed according to these criteria. Biopsies are needed for proving fungal infections but are often not feasible because of the high-risk of complications. Serum markers, such as β-d-glucan value, are often used but the usefulness of these markers is still controversial [18]. Prophylaxis for fungal infection is also influenced by the decisions of the attending physicians. As per our hospital policy, we used fluconazole as the first-line prophylactic agent for fungal infection during the treatment of acute leukemia, but some patients were prescribed mold-active agents, such as itraconazole, voriconazole, echinocandins, and amphotericin B. Prospective study design might be preferable for standardizing the criteria for diagnosis and prophylaxis of fungal infections.

## Conclusions

We found that the preventive effect of the LAF isolation system declined with long-term use. We should reconsider and establish the maintenance and assessment procedures of air quality in the ward, appropriate isolation strategies of immunocompromised patients, and fungal infection prophylaxis, especially in patients who are highly exposed to environmental factors.

## Abbreviations

HR: Hazard ratio
LAF: Laminar air flow
